# Clear surgical margins as a prognostic indicator for disease recurrence, with no impact on survival rates in patients with myxofibrosarcoma

**DOI:** 10.1038/s41598-024-63035-6

**Published:** 2024-05-28

**Authors:** Tomáš Tomáš, Vasileios Apostolopoulos, Lukáš Pazourek, Marián Kubíček, Iva Staniczková Zambo, Dagmar Adámková, Pavel Šlampa, Michal Mahdal

**Affiliations:** 1 First Department of Orthopaedic Surgery, St. Anne’s University Hospital, 60200 Brno, Czech Republic; 2 Faculty of Medicine, Masaryk University, 62500 Brno, Czech Republic; 3 First Pathology Department, St. Anne’s University Hospital, 60200 Brno, Czech Republic; 4 Clinic of Comprehensive Cancer Care, Masaryk Memorial Cancer Institute, 60200 Brno, Czech Republic; 5 Department of Radiation Oncology, Masaryk Memorial Cancer Institute, 60200 Brno, Czech Republic

**Keywords:** Surgical oncology, Sarcoma

## Abstract

Myxofibrosarcoma presents an infiltrating growth pattern that results in a high tendency for local recurrence. Clear margin resection is challenging because of microscopic infiltration. The purpose of the present study was to analyze the overall and disease-free survival rates of patients with myxofibrosarcoma and the prognostic factors that determine both survival and disease recurrence. Among the 111 patients included in our study, the 5-year overall survival rate was 65.5%. An age of more than 65 years (hazard ratio [HR] 1.9 [95% confidence interval (CI) 1.4–5.6]; p < 0.001), a tumor size of more than 5 cm (HR 2.8 [95% CI 0.9–8.1]; p = 0.049) and the G3 tumor grade (HR 14.1 [95% CI 2.1–105.0]; p < 0.001) negatively affected overall survival. The 5-year recurrence-free survival rate was 49.4%. R1/R2-type resection (HR 2.4 [95% CI 1.0–5.6]; p = 0.048) had a detrimental effect on tumor recurrence. Clear margins had a positive impact on recurrence-free survival, but did not significantly affect overall patient survival, suggesting that other factors may play a more significant role in determining patient outcomes. A surgical margin of 2 mm was not sufficient to significantly influence the incidence of recurrence. Consequently, a wider surgical margin may be necessary to reduce the risk of myxofibrosarcoma recurrence.

## Introduction

Myxofibrosarcoma is a type of soft-tissue sarcomas, formerly classified as the myxoid variant of malignant fibrous histiocytoma^1,2^. Histologically, myxofibrosarcoma combines hypocellular myxoid and a fibroblastic nodular appearance^3^. Myxofibrosarcoma presents an infiltrating character that leads to extension along the fascia and growth into the subcutaneous tissue, skeletal muscle, and bone, often with misleading boundaries^4,5^. This infiltrative growth pattern results in a high tendency for local recurrence as clear margin resection is challenging because of microscopic infiltration^6–8^. The incidence of myxofibrosarcoma recurrence is reported to be higher compared with the other types of soft-tissue sarcomas^4,9–11^. On the contrary, the reported survival rates of patients with myxofibrosarcoma are relatively high compared to other sarcomas^12,13^.

The purpose of the present study was to retrospectively analyze all patients diagnosed with myxofibrosarcoma in our institution from 2010 to 2022. We focused on analyzing the overall and disease-free survival rates of patients and the prognostic factors that determine both survival and disease recurrence.

## Methods

### Patient data

This retrospective study analyzed the records of 111 patients with histologically diagnosed myxofibrosarcoma, treated between 2010 and 2022. Of them, 106 patients were treated surgically. In all of the cases, the attempt was to achieve a complete, clear margin resection. When the presence of the ‘tail sign’ was detected on preoperative magnetic resonance imaging, the aim was to include it into the resection specimen during the surgical procedure. Five patients had an inoperable tumor in the extremities with multiple lung metastases. These patients were included in the survival analysis and excluded from the disease and local recurrence analyses. Thirty-five patients were initially surgically treated in a non-sarcoma center with an unplanned excision of myxofibrosarcoma. Of them, twelve patients did not have preoperative magnetic resonance imaging (MRI). The patients’ age, sex, tumor grading, tumor size, anatomical location, tumor invasion, adjuvant and neoadjuvant therapy, type of resection, and surgical margins were recorded (Table 1). The dataset included 55 female and 56 male patients with a mean age at diagnosis of 63.3 ± 14.2 years. The mean follow-up was 3.6 ± 3.3 years. Postoperative routine follow-up evaluation was performed every 3 months for the first 2 years, every 6 months for the next 3 years, and then annually. Each follow-up evaluation included clinical examination and imaging methods. Most of the patients (41.4%) were preoperatively diagnosed with a G3-classified myxofibrosarcoma. The most frequent tumor site was the thigh (19.8%) and the tumor invasion was subfascial (deep) in 62.2% of the cases (Table 2). The closest margin was measured by an experienced pathologist, and based on the margin the resection was categorized according to residual tumor classification. The examination of prognostic factors was conducted based on the records from the initial surgical procedure.

**Table 1:** Sample characteristics.

Characteristic	Value
Number of patients	111
Age at inclusion (years), mean ± standard deviation	63.3 ± 14.2
Sex, n (%)	
Female	55 (49.5%)
Male	56 (50.5%)
Follow-up (years)	3.6 ± 3.3
Grading, n (%)	
G1	34 (30.6%)
G2	31 (27.9%)
G3	46 (41.4%)
Size (maximum length in mm), mean ± standard deviation	93.4 ± 52.8
Tumor invasion, n (%)	
Superficial	42 (37.8%)
Deep	69 (62.2%)
Radiotherapy, n (%)	
No	62 (55.9%)
Neoadjuvant	11 (9.9%)
Adjuvant	36 (32.4%)
Both	2 (1.8%)
Chemotherapy, n (%)	
No	97 (87.4%)
Neoadjuvant	4 (3.6%)
Adjuvant	4 (3.6%)
Both	1 (0.9%)
Palliative	5 (4.5%)
Site of initial treatment, n (%)	
Sarcoma center	76 (68.5%)
Non-sarcoma center	35 (31.5%)

**Table 2:** Sample characteristics: anatomical site.

Characteristic	Value
Anatomical site, n (%)	
Axilla	6 (5.4%)
Arm	8 (7.2%)
Forearm	10 (9.0%)
Hand	5 (4.5%)
Gluteal	9 (8.1%)
Inguinal	7 (6.3%)
Thigh	33 (29.7%)
Knee	5 (4.5%)
Calf	16 (14.4%)
Foot	4 (3.6%)
Other	8 (7.2%)

After presenting all cases to the multidisciplinary Musculoskeletal Tumor Committee, the treatment management was determined on an individual basis and adhered to the standards of evidence-based medicine. Radiotherapy (dosage range: 50–65 Gy) was recommended for cases at high risk of local recurrence, specifically those with G3 tumors and a size exceeding 5 cm. Furthermore, radiotherapy was advised in situations where the radicality of the potential surgical resection was uncertain due to anatomical constraints. Currently, there is a clear preference for administering preoperative radiotherapy in cases where there is a potential postoperative indication. In this cohort, chemotherapy was administered to patients with an exceptionally high risk of disease recurrence, where the estimated 10-year survival rate was below 60% as per prognostic nomograms. This study was conducted by adhering to the guidelines of the Declaration of Helsinki, and consent was obtained from all patients involved in the study.

### Evaluation

The study endpoints were overall survival, recurrence-free survival, and prognostic factors determining survival and disease recurrence. Overall survival refers to tumor-specific survival and does not include deaths from natural causes. It was calculated starting at the date of the definitive histological examination result until the date of death. Recurrence-free survival refers to the period after the initial treatment of myxofibrosarcoma during which the patient remains free of local or distant recurrence. It was calculated starting at the date of the definitive histological examination result until the date of recurrence capture. Based on previous studies, prognostic factors determining survival, disease recurrence, and local recurrence were examined: age, sex, tumor size (more or less than 5 cm), tumor invasion (deep or superficial), grade, resection type (R0 or R1/2), resection margin (more or less than 2 mm), radiotherapy, and site of surgery (sarcoma center or non-sarcoma center)^5,14,15^.

### Statistical analysis

Descriptive analysis of the overall survival estimation was based on Kaplan–Meier models in an examined period of 100 months. The log-rank test was used to compare differences in survival between groups. Descriptive analysis of the recurrence-free survival estimation was based on Kaplan–Meier models in an examined period of 60 months. The log-rank test was used to compare differences between groups. Cox proportional hazard models were used to identify independent prognostic factors influencing overall and recurrence-free survival. A multivariate Cox regression analysis was performed to investigate the prognostic factors for overall survival and local recurrence A two-sided p-value below the 0.05 was considered to be the threshold for statistical significance. All statistical analyses were performed by using R software (version 4.0.5) in the RStudio development environment (Bell Laboratories, Murray Hill, NJ, USA).

### Statement of ethics

The study protocol was approved by the St. Anne’s University Hospital Ethics Committee (no: EK-FNUSA-01/2024).

### Informed consent

Informed consent was obtained from all subjects involved in the study.

## Results

### Overall survival

**Figure 1:**
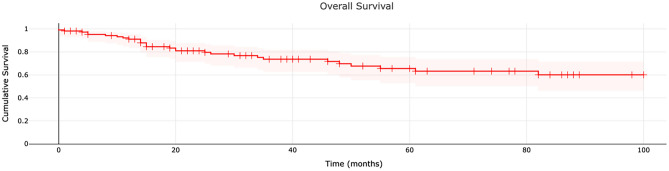
Overall survival of patients with myxofibrosarcoma.

At a median follow-up of 43.3 (± 39.2) months, the 1-year overall survival rate was 91.1% (number at risk 89). The 3 and 5-year overall survival rates were 73.6% (number at risk 47) and 65.5% (number at risk 29), respectively. The 8-year overall survival rate was 60.0% (number at risk 13) (Fig. 1).

### Prognostic factors for survival

From the investigated factors, an age of more than 65 years (hazard ratio [HR] 1.9 [95% CI 1.4–5.6]; p < 0.001), a tumor size of more than 5 cm (HR 2.8 [95% CI 0.9–8.1]; p = 0.049), and the G3 tumor grade (HR 14.1 [95% CI 2.1–105.0]; p < 0.001) negatively affected overall survival. The G1 tumor grade was associated with better overall survival (HR 0.1 [95% CI 0.0–0.6]; p = 0.017). The type of resection in terms of residual tumor classification did not influence overall survival (HR 1.1 [95% CI 0.4–2.7]; p = 0.844). In addition, a margin of more than 2 mm did not influence overall survival (HR 0.8 [95% CI 0.3–1.9]; p = 0.555) (Table 3).

**Table 3:** Prognostic factors for survival of patients with myxofibrosarcoma.

Variable	Number in the exposed group	Hazard ratio	95% confidence interval	p-value
Age				
< 65 years	54	Ref		
≥ 65 years	57	1.9	1.4–5.6	0.001
Sex				
Male	56	Ref		
Female	55	1.1	0.5–2.6	0.785
Tumor size				
≤ 5 cm	32	Ref		
> 5 cm	79	2.8	0.9–8.1	0.049
Tumor invasion				
Superficial	42	Ref		
Deep	69	1.5	0.6–3.7	0.381
Grade				
G1	34	0.1	0.0–0.6	0.017
G2	31	3.8	0.4–10.8	0.174
G3	46	14.1	2.1–105.0	0.001
Surgery				
R0	71	Ref		
R1/R2	35	1.1	0.4–2.7	0.844
Radiotherapy				
No	62	Ref		
Yes	49	1.3	0.5–2.9	0.603
Site of primary surgery				
Sarcoma center	71	Ref		
Non-sarcoma center	35	0.6	0.2–1.7	0.374
Resection margins				
< 2 mm	75	Ref		
≥ 2 mm	31	0.8	0.3–1.9	0.555

### Overall survival of patients with tumors more than 5 cm in size and dependence on anatomical site

**Figure 2:**
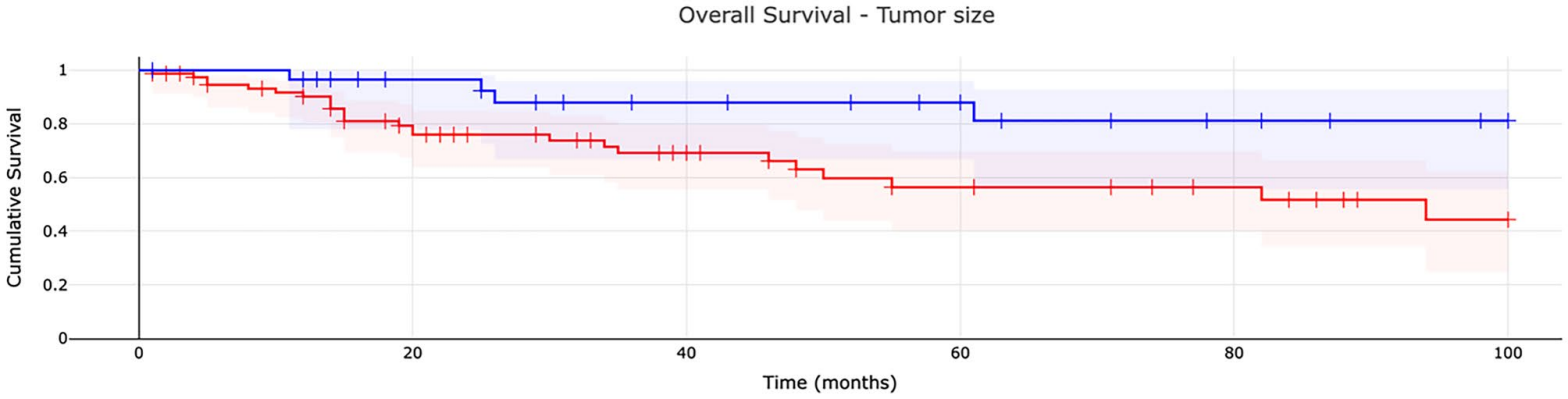
Overall survival of patients with myxofibrosarcoma: tumor size analysis. The survival rates are shown for patients with tumors more than 5 cm in size (red) and less than 5 cm in size (blue).

The 1-, 5-, and 8-year overall survival rates of patients with tumors more than 5 cm in size were 90.1% (number at risk 62), 56.4% (number at risk 35), and 44.3% (number at risk 11), respectively. Those rates were significantly lower compared with patients with tumors less than 5 cm in size: 96.5% (number at risk 27) at 1 year, 87.9% (number at risk 12) at 5 years, and 81.2% (number at risk 2) at 8 years (p = 0.016) (Fig. 2).

In addition, we investigated whether anatomical site affects overall survival. Despite the low incidence of axillary tumors (n = 6), this location negatively influenced overall survival (HR 16.9 [95% CI 1.9–151.5]; p = 0.011). The presence of tumors at other anatomical sites did not impact overall survival.

### Recurrence-free survival

**Figure 3:**
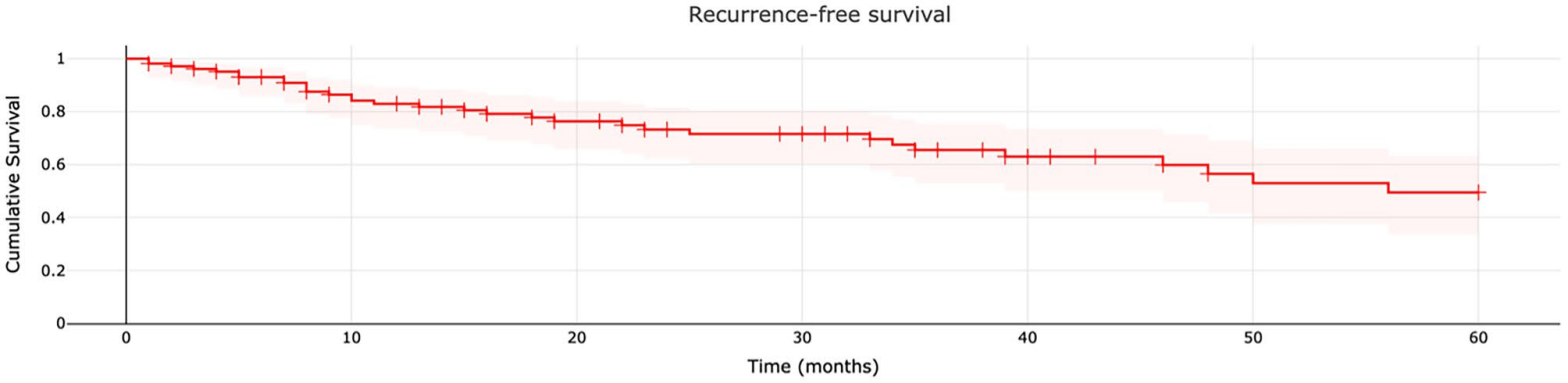
Recurrence-free survival of patients with myxofibrosarcoma.

The 1-, 3-, and 5-year recurrence-free survival rates were 82.9% (number at risk 77), 65.4% (number at risk 35), and 49.4% (number at risk 20), respectively (Fig. 3). Recurrence occurred in 32 cases, and the mean time to capture was 18.5 ± 15.9 months. Of these 32 cases, 25 were local recurrences (mean time to capture 16.8 ± 15.7 months) and 7 were distant recurrences (mean time to capture 24.6 ± 16.4 months).

### Prognostic factors for disease recurrence

The type of resection performed, in terms of residual tumor classification, had a significant impact on tumor recurrence (HR 2.4 [95% CI 1.0–5.6]; p = 0.048). Furthermore, patients aged 65 and older were at a higher risk of disease recurrence (HR 3.2 [95% CI 1.3–7.8]; p = 0.009). There was a negative trend when a patient had the first operation in a non-sarcoma center, but this effect was not confirmed (HR 2.1 [95% CI 0.9–4.9]; p = 0.096). On the other hand, there was a positive trend with a margin of more than 2 mm (HR 0.6 [95% CI 0.2–1.5]; p = 0.275) (Table 4).

**Table 4:** Prognostic factors for disease recurrence in patients with myxofibrosarcoma.

Variable	Number in the exposed group	Hazard ratio	95% confidence interval	p-value
Age				
< 65 years	54	Ref		
≥ 65 years	52	3.2	1.3–7.8	0.009
Sex				
Male	54	Ref		
Female	52	1.5	0.7–3.4	0.331
Tumor size				
≤ 5 cm	32	Ref		
> 5 cm	74	1.1	0.4–2.6	0.875
Tumor invasion				
Superficial	41	Ref		
Deep	65	0.9	0.4–2.1	0.826
Grade				
G1	34	0.4	0.1–1.0	0.058
G2	27	1.7	0.4–5.6	0.091
G3	45	5.2	0.7–13.2	0.068
Surgery				
R0	71	Ref		
R1/R2	35	2.4	1.0–5.6	0.048
Radiotherapy				
No	58	Ref		
Yes	48	1.1	0.5–2.5	0.828
Site of primary surgery				
Sarcoma center	71	Ref		
Non-sarcoma center	35	2.1	0.9–4.9	0.096
Resection margins				
< 2 mm	75	Ref		
≥ 2 mm	31	0.6	0.2–1.5	0.275

### Recurrence-free survival rates of patients with clear margin resection and dependence on the anatomical site

**Figure 4:**
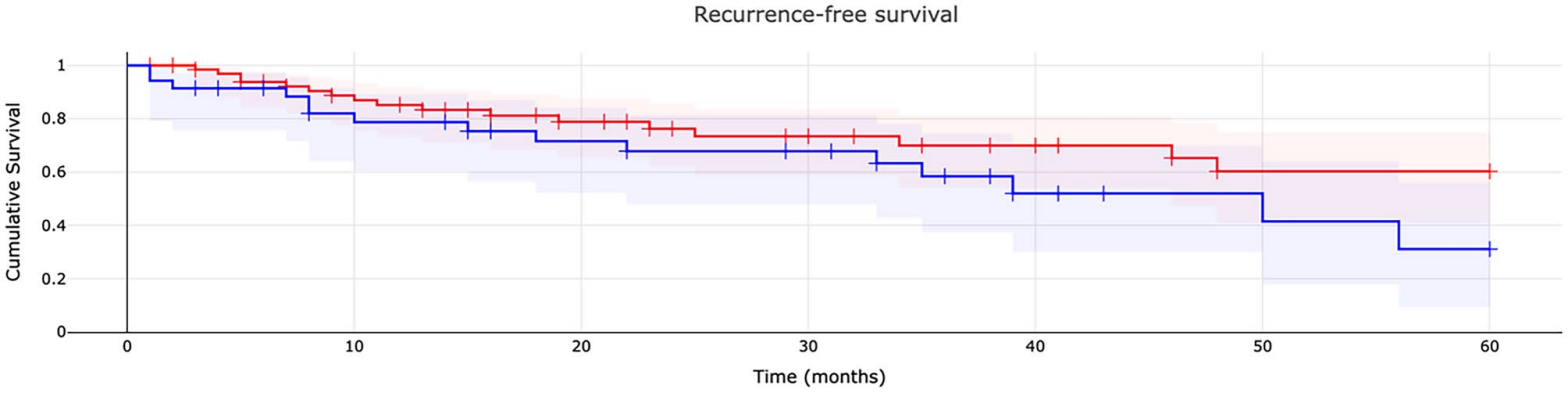
Recurrence-free survival of patients with myxofibrosarcoma: type of resection (R0/R1). Cumulative survival is shown for patients with clear margin resection (red) and patients with microscopic infiltration (blue).

The 1-, 3-, and 5-year recurrence-free survival rates of patients with clear margin resection were 85.1% (number at risk 54), 69.9% (number at risk 23), and 60.2% (number at risk 16), respectively. These rates were significantly higher compared with patients with microscopic infiltration: 78.6% (number at risk 23) at 1 year, 58.3% (number at risk 12) at 3 years, and 31.1% (number at risk 4) at 5 years (p = 0.120) (Fig. 4).

In addition, we investigated whether the anatomical site affects tumor recurrence. Despite the low incidence of axillary tumors (n = 6), this location was a risk factor for recurrence (HR 13.3 [95% CI 1.5–119.4]; p = 0.02). On the contrary, when myxofibrosarcoma occurred in the thigh area, tumor recurrence occurred less frequently (HR 0.2 [95% CI 0.0–0.6]; p = 0.006) (Table 5).

**Table 5:** Prognostic factors for disease recurrence in patients with myxofibrosarcoma: anatomical site.

Variable	Number of recurrences	Hazard ratio	95% confidence interval	p-value
Anatomical location				
Axilla	6	13.3	1.5–119.4	0.020
Arm	1	1.5	0.3–6.8	0.576
Forearm	4	1.7	0.5–6.6	0.418
Hand	3	1.5	0.3–6.8	0.576
Gluteal	1	0.3	0.0–2.4	0.247
Inguinal	2	1.0	0.2–5.4	0.987
Thigh	3	0.2	0.0–0.6	0.006
Knee	2	1.7	0.3–10.6	0.576
Calf	7	2.3	0.8–6.8	0.134

### Prognostic factors for local recurrence

Of the investigated factors, the type of resection in terms of residual tumor classification (HR 3.6 [95% CI 1.4–9.2]; p = 0.006) and an age of more than 65 years (HR 2.6 [95% CI 1.1–5.9]; p = 0.024) negatively affected local recurrence. There was a negative trend for local recurrence when a patient had the first operation in a non-sarcoma center (HR 1.9 [95% CI 0.7–4.7]; p = 0.185). On the other hand, G1 tumors locally recurred less frequently (HR 0.2 [95% CI 0.1–0.7]; p = 0.011) (Table 6).

**Table 6:** Prognostic factors for local recurrence in patients with myxofibrosarcoma.

Variable	Number in the exposed group	Hazard ratio	95% confidence interval	p-value
Age				
< 65 years	54	Ref		
≥ 65 years	52	2.6	1.1–5.9	0.024
Sex				
Male	54	Ref		
Female	52	1.8	0.7–4.4	0.213
Tumor size				
≤ 5 cm	32	Ref		
> 5 cm	74	0.7	0.3–1.6	0.417
Tumor invasion				
Superficial	41	Ref		
Deep	65	0.9	0.4–2.3	0.876
Grade				
G1	34	0.2	0.1–0.7	0.011
G2	27	1.9	0.2–4.9	0.213
G3	45	4.5	1.3–15.9	0.018
Surgery				
R0	71	Ref		
R1/R2	35	3.6	1.4–9.2	0.006
Radiotherapy				
No	58	Ref		
Yes	48	1.8	0.7–4.3	0.220
Site of primary surgery				
Sarcoma center	71	Ref		
Non-sarcoma center	35	1.9	0.7–4.7	0.185
Resection margins				
< 2 mm	75	Ref		
≥ 2 mm	31	0.7	0.3–2.0	0.561

### Multivariate Cox regression analysis of prognostic factors for overall survival and local recurrence

A multivariate Cox regression analysis was conducted to investigate the prognostic factors for overall survival and local recurrence (Table 7). The findings revealed a significant association between G3 tumor grade and poorer overall survival (HR 16.1 [95% CI 2.1–125.1]; p = 0.008) as well as an increased risk of local recurrence (HR 4.2 [95% CI 1.1–15.9]; p = 0.033). Although not statistically significant, individuals aged over 65 years showed a negative trend for both survival (HR 2.1 [95% CI 0.9–4.7]; p = 0.079) and local recurrence (HR 1.9 [95% CI 0.8–4.7]; p = 0.156). A surgical margin of 2 mm was insufficient to influence overall survival (HR 0.7 [95% CI 0.2–2.3]; p = 0.631) or local recurrence (HR 1.1 [95% CI 0.3–3.7]; p = 0.888).

**Table 7:** Multivariate Cox regression analysis of prognostic factors for overall survival and local recurrence.

Variable	Survival			Local recurrence		
	Hazard ratio	95% confidence interval	p-value	Hazard ratio	95% confidence interval	p-value
Age						
< 65 years	Ref			Ref		
≥ 65 years	2.1	0.9–4.7	0.079	1.9	0.8–4.7	0.156
Tumor size						
≤ 5 cm	Ref			Ref		
> 5 cm	1.7	0.5–5.9	0.367	0.5	0.2–1.2	0.110
Resection margins						
< 2 mm	Ref			Ref		
≥ 2 mm	0.7	0.2–2.3	0.631	1.1	0.3–3.7	0.888
Grade						
G1/2	Ref			Ref		
G3	16.1	2.1–125.1	0.008	4.2	1.1–15.9	0.033

## Discussion

Myxofibrosarcoma is a frequently encountered form of soft tissue sarcoma, known for its complexity in treatment and unsatisfactory clinical outcomes^8,16^. Given the prevalence of this condition, it has become crucial to explore survival rates and prognostic factors in depth, particularly from a specialized sarcoma center. To address this issue, we conducted a comprehensive analysis on the survival of patients, disease-free rates, and prognostic factors within the context of our specialized sarcoma center. By illuminating these aspects, we have contributed valuable insights to the understanding and management of myxofibrosarcoma.

In our cohort of 111 patients with myxofibrosarcoma surgically treated at our institution, the 1-, 3-, and 5-year overall survival rates were 91.1%, 73.6%, and 65.5%, respectively. The recorded 8-year overall survival rate was 60.0%, and the number at risk was only 13 patients because of the loss to follow-up. A similar 5-year overall survival rate (67.7%) was reported in the clinical data of patients with myxofibrosarcoma by using a nationwide database from the Netherlands Cancer Registry^15^. The reported 5-year overall survival rate has ranged from 61 to 71% in other single institution cohorts^9,17,18^. Sanfilippo et al.^12^ recorded a slightly higher 5-year survival rate of 77%^12^. Those results confirm that myxofibrosarcoma exhibits better disease-specific survival than other soft tissue sarcoma subtypes.

Tumor size, histological grade, and the axilla site were recorded as negative prognostic factors for the patients' survival. Surprisingly, the status of surgical margins in the primary resection was not shown to be an independent predictor of survival. This finding contradicts previous studies^9,12,15^. Even a resection margin of 2 mm did not influence the mortality. A possible explanation could be the intake of patients who were previously treated at external, non-specialized surgical institutions. In most of those cases, the resection resulted in residual disease or microscopical infiltration, but patients underwent a re-resection with wide margins at our institution in a timely fashion. However, Sambri et al. recorded similar survival in patients with R0 and R1 surgical margins^19^. It is challenging to excise axillary tumors completely and to prevent disease relapse. Our cohort presented a high mortality of patients with a prevalence of myxofibrosarcoma at this anatomical site. In the tumor-specific survival analysis, patients with tumors smaller than 5 cm exhibited a 24.8% higher 5-year survival rate compared with patients with tumors larger than 5 cm. Tumors less than 5 cm in size are often misdiagnosed as benign. Studies have reported an increased risk of unplanned excisions and often inadequate margins^20–22^. On the contrary, patients with unplanned excision did not present worse survival, which can be described because of the smaller tumor size.

The recurrence of myxofibrosarcoma is higher compared with other types of soft tissue sarcomas^4,13^. The recurrence-free rates described in the literature range from 75 to 50%^19,23^. In our study, we observed a recurrence-free rate of 49.4% at the 5-year mark after the initial surgical procedure. It is important to note that this cohort included patients who were initially treated at external non-specialized surgical institutions. We found that patients who were initially treated at these institutions had a higher risk of recurrence, with an HR of 2.1, but this factor was not statistically confirmed. Out of the 35 patients in this group, only 12 did not experience recurrence of the disease. Among the recurrences that were recorded (n = 32), the majority of them were local recurrences (n = 25), while 7 cases were classified as distant recurrences. It is noteworthy that there was a difference in the mean time to capture these recurrences. Distant recurrences were registered later, with an average time of 24.6 ± 16.4 months, compared with local recurrences. The incidence of distant recurrences in the literature is slightly higher and ranges from 15 to 38%^12,18,24^. A recent study described a 28% distant recurrence rate with a median time to recurrence of 15.3 months^15^.

In our analysis of prognostic factors, we found that the type of surgical resection (R0 vs R1) was the only factor that showed a statistically significant impact on disease recurrence. In the available literature, tumor grading, size, and unplanned excision were noted to influence the incidence of recurrence^15,19,23,24^. We observed that a resection margin of 2 mm was not sufficient to influence the incidence of recurrence. Of a total of 6 patients with axillary tumors, 5 experienced a recurrence of the disease. In most cases, there was microscopic infiltration (R1) of the resection margin, with 4 cases showing this characteristic. This suggests that achieving clear margins during surgery is crucial in preventing recurrence. On the other hand, the thigh site was found to be a protective factor against recurrence (HR 0.2). Out of the 33 thigh sites examined, only 6 had R1 resection, indicating that in most cases, clear margins were achieved. Additionally, the margins at the thigh site were generally greater compared with other sites, with a mean margin of 3.5 mm. This suggests that a larger margin may contribute to reducing the risk of recurrence in this location. In the type of resection-specific analysis, the recurrence-free survival rate of patients with clear margin resection (60.2%) was higher compared with patients with microscopic infiltration (31.1%) at 5 years after the initial surgical procedure. The effect of radiotherapy is still controversial. The local recurrence rates in patients treated with neoadjuvant and/or adjuvant radiotherapy are comparable to those who did not receive radiotherapy^24–26^. However, high-risk patients were treated with radiotherapy, and this variability may have affected the results. Considering the limitation, in our cohort, the adjuvant radiotherapy had no obvious effect on local control. Chemotherapy was only administered in rare cases, specifically to patients who had an exceptionally high risk of disease recurrence (n = 9) and to 5 patients with inoperable tumors.

There are several limitations to consider in this retrospective study on myxofibrosarcoma. Firstly, it is important to note that the data were collected from a single center. While this can provide valuable insights into the specific population studied, it may limit the generalizability of the findings. Additionally, the mean follow-up duration in this study may not have been sufficiently long to capture late recurrences or overall survival outcomes. Myxofibrosarcoma is known for its potential for late recurrences, so future studies with longer follow-up periods would be beneficial to better understand the long-term outcomes and survival rates.

Furthermore, we analyzed factors leading to both local and distant recurrences, despite surgical margins mainly influencing local recurrences. In the anatomical location analyses, given the limited number of patients, it is challenging to generalize our results obtained. However, there was a tendency of poorer outcomes in the axilla. Finally, it is worth noting that the study included patients who were initially treated at external, non-specialized surgical institutions. This could potentially affect the clarity of the outcome data and introduce bias in the results. However, we analyzed the impact of this factor and tried to account for this potential limitation.

## Conclusions

The present study examined the survival rates of patients with myxofibrosarcoma and evaluated the factors that contribute to both survival and disease recurrence outcomes. A positive resection margin was associated with adverse prognosis in local recurrence, while this element had no impact on survival. Age, tumor size, and high grades were associated with adverse survival. A surgical margin of 2 mm did not significantly influence the incidence of recurrence. This suggests that a wider surgical margin may be necessary to reduce the risk of tumor recurrence. The successful diagnosis and treatment of myxofibrosarcoma requires a comprehensive and multidisciplinary approach, which is best provided by specialized sarcoma centers. These findings provide valuable insights into the factors that influence survival and recurrence outcomes in patients with myxofibrosarcoma.

## Data Availability

The data that support the findings of this study are available from the corresponding author upon reasonable request.
